# Narcolepsy and Psychiatric Disorders: Comorbidities or Shared Pathophysiology?

**DOI:** 10.3390/medsci6010016

**Published:** 2018-02-15

**Authors:** Anne Marie Morse, Kothare Sanjeev

**Affiliations:** 1 Division of Child Neurology and Sleep Medicine, Geisinger Medical Center, Danville, PA 17820, USA; amorse@geisinger.edu; 2 Division of Pediatric Neurology, Pediatric Sleep Program (Neurology), Department of Pediatrics, Cohen Children’s Medical Center, Lake Success, NY 11042, USA

**Keywords:** narcolepsy, schizophrenia, attention deficit hyperactivity disorder, depression, anxiety, psychiatric disorders

## Abstract

Narcolepsy and psychiatric disorders have a significant but unrecognized relationship, which is an area of evolving interest, but unfortunately, the association is poorly understood. It is not uncommon for the two to occur co-morbidly. However, narcolepsy is frequently misdiagnosed initially as a psychiatric condition, contributing to the protracted time to accurate diagnosis and treatment. Narcolepsy is a disabling neurodegenerative condition that carries a high risk for development of social and occupational dysfunction. Deterioration in function may lead to the secondary development of psychiatric symptoms. Inversely, the development of psychiatric symptoms can lead to the deterioration in function and quality of life. The overlap in pharmaceutical intervention may further enhance the difficulty to distinguish between diagnoses. Comprehensive care for patients with narcolepsy should include surveillance for psychiatric illness and appropriate treatment when necessary. Further research is necessary to better understand the underlying pathophysiology between psychiatric disease and narcolepsy.

## 1. Introduction

Narcolepsy is a disabling neurodegenerative condition that is characterized by the pentad features of excessive daytime sleepiness (EDS), sleep fragmentation, sleep related hallucinations, sleep paralysis, and cataplexy; brief episodes of loss of tone frequently provoked by strong emotions. Instability in the transition between wakefulness and rapid eye movement (REM) sleep causes these symptoms. 

Diagnosis is generally made based on the presence of EDS and findings of an average sleep latency of ≤8 and the presence of two or more sleep onset REM periods (SOREMPs) on sleep testing. SOREMPs are the presence of REM sleep within 15 minutes of sleep onset, as opposed to the typical cycle taking about 90–120 min. Alternatively, it may be diagnosed by evaluating cerebrospinal fluid (CSF) hypocretin (HRT), which is found to be low in narcolepsy type 1. It is estimated to affect about 1 in 2000 individuals and frequently can take as long as 8–10 year to be accurately diagnosed [1].

Narcolepsy has long been described to have a high co-morbidity for psychiatric disease [2], which is frequently quoted as the cause for delay in diagnosis. The underpinnings of the development of psychiatric symptoms, however, remain unclear. It has been suggested that psychiatric symptoms are either a result of the chronic disabling nature of the disease or it may represent a “shared pathophysiology” or a combination of both. 

**Figure 1:**
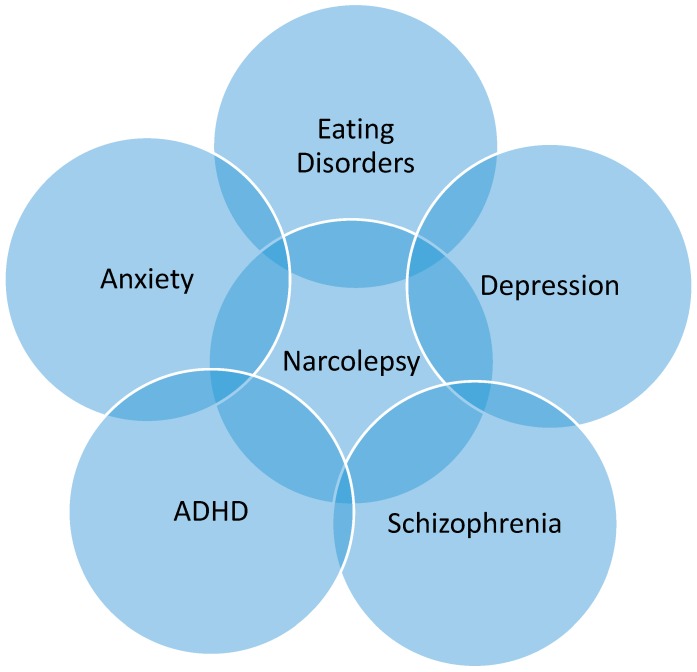
Venn Diagram of the overlapping relationship between highlighting the intimate relationship between psychiatric disorders and narcolepsy [3,4,5,6,7,8,9,10,11,12]. ADHD: attention deficit hyperactivity disorder.

Improved familiarity with psychiatric illnesses that may share similar features to narcolepsy or may be comorbid (Figure 1) may improve therapeutic outcomes. Consideration of narcolepsy as a part of the differential diagnosis for psychiatric disease may reduce time to diagnosis. Additionally, regular assessment for co-occurring psychiatric disorders in narcolepsy patients may also improve quality of life and functionality. 

## 2. Attention Deficit Hyperactivity Disorder 

Attention deficit hyperactivity disorder (ADHD) is characterized by symptoms of inattention, impulsivity and hyperactivity [13]. Many clinicians perceive ADHD to be the antithesis of narcolepsy; however, there is a significant clinical similarity. Historically, there has even been the suggestion for various overlap syndromes, such as Syndrome Z and Primary Disorder of Vigilance, which were defined by a combination of narcolepsy and ADHD symptoms [14,15]. 

Recently, there is increasing evidence that sleep dysfunction is intimately related to the development of attention deficit hyperactivity disorder (ADHD). Restricted, dysfunctional, or fragmented sleep may precipitate ADHD features [16,17]. On the other hand, problems with sleep may represent an intrinsic component of ADHD [18]. Individuals with ADHD have an increased association with restless legs syndrome/periodic limb movements in sleep (RLS/PLMS), obstructive sleep apnea/snoring, rhythmic movement disorder (body rocking and head banging), and parasomnias [9,17,18]. The presence of ADHD symptoms in children and adolescents with narcolepsy were found to be about two-fold higher than in controls [9]. Retrospectively, adults with narcolepsy had been found to have a much greater likelihood of having a diagnosis of ADHD in childhood compared to controls [11]. 

Alternatively, these features may be related to or even confused with the sense of cognitive impairments such as mental fog and difficulty thinking. Cognitive features, such as mental fogginess, have been found to be among the most significant symptoms affecting the daily life of patients with narcolepsy [19]. Hyperactivity seen in ADHD may, in fact, be a compensatory response for individuals who are under-aroused or sleepy [11]. ADHD symptoms have been shown to contribute to poor quality of life and increased frequency of depressive symptoms [20] similar to narcolepsy. 

**Figure 2:**
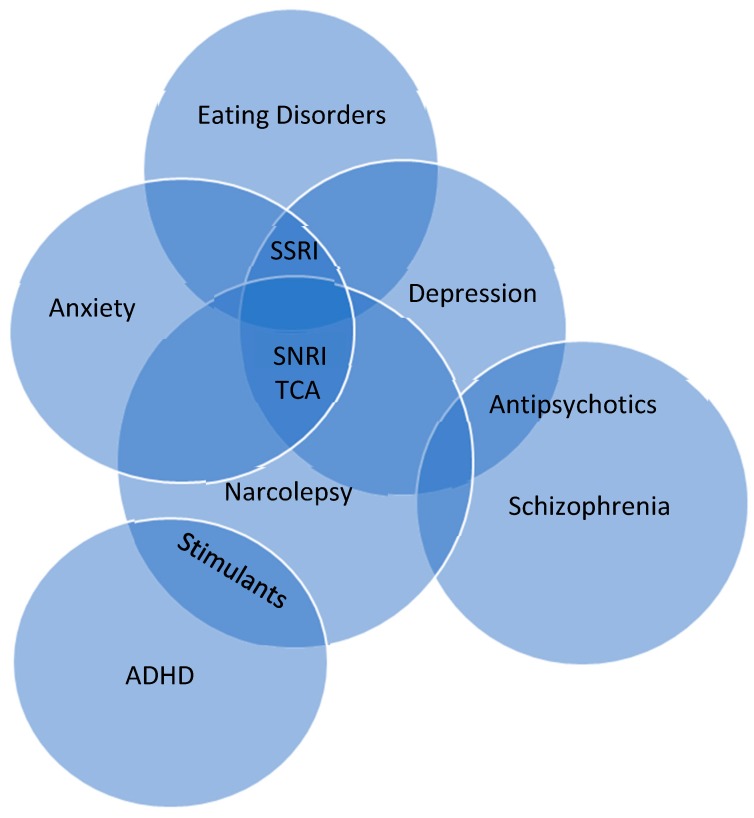
Venn diagram of the overlapping relationship in pharmaceutical treatment for narcolepsy and select psychiatric disorders [4,5,6,9,10,11,24,25]. Overlapping benefit of pharmaceutical treatment is found in most treatments, except antipsychotics, which can exacerbate symptoms of narcolepsy. SNRI: Serotonin norepinephrine reuptake inhibitor; TCA: tricyclic antidepressent; SSRI: selective serotonin reuptake inhibitor; ADHD: attention deficit hyperactivity disorder.

Pharmaceutical interventions for ADHD has overlap (Figure 2) with treatment used in narcolepsy for excessive daytime sleepiness, potentially masking the clinical features of narcolepsy [21,22]. There has been consideration for hypocretin deficiency to be causative for the shared symptoms in narcolepsy and ADHD. However, ADHD symptoms have been found in narcolepsy type 1 and type 2, suggesting that hypocretin deficiency may be unrelated to shared symptomatology. This has been further confirmed with CSF hypocretin evaluation [9]. Therefore, symptoms of EDS, fatigue, and sleep fragmentation may be the cause for ADHD symptoms, which may also explain similar findings in other sleep wake disorders [23]. 

## 3. Depression

Depressed mood is the most commonly described psychiatric symptom in narcolepsy literature [26,27,28]. Studies evaluating narcoleptic patients with self-reported questionnaires have found up to 57% suffered from depression [26,27]. It is frequently suggested that this is due to the significant overlap in symptoms, such as disordered nocturnal sleep, social withdrawal, impaired attention, fatigue, and weight gain (Table 1). However, when excluding symptoms that may represent overlap, a higher level of depressive symptoms is still present in patients with narcolepsy, including features of anhedonia, pathological guilt, and crying [28]. Depressed mood and sleepiness have been found to be the main limiting factors in maintaining attention in patients with narcolepsy [29]. Additionally, depression has been found to be a major independent risk factor for impaired quality of life [30]. 

The chronicity and debilitating nature of narcolepsy may provide the psychological substrate for development of depression. However, Lee et al. identified more than 50% of patients who had narcolepsy and comorbid depression had been diagnosed with depression prior to narcolepsy [10]. Therefore, a shared pathophysiology related to hypocretin deficiency should be considered. Recent research has suggested HRT deficiency impedes appropriate emotional input processing within the amygdala [31]. Further support for this concept was found in the post-mortem evaluation of CSF HRT in depressed patients who completed suicide, which also demonstrated lowered levels of HRT [32]. However, these findings have been inconsistent in other studies [33].

## 4. Anxiety Disorders

Anxiety disorders are receiving increasing attention as co-morbidity in narcolepsy, but references are still relatively scarce. Anxiety disorders, such as panic attacks and social phobias, have been reported in as many as 53% of patients with narcolepsy [6]. The time course of development for specific anxiety disorders has been suggested to vary by type. For instance, obsessive compulsive disorder and social phobia are more frequently present before the diagnosis of narcolepsy, while panic disorder or simple phobia occur afterward [34]. It has been suggested that these symptoms may be a result of a perceived loss of personal control, such as is experienced with a cataplectic event. Alternatively, it may be related to a fragmented perception of reality due to experienced hallucinations [7].

## 5. Eating Disorders

Patient with narcolepsy are frequently overweight [3]. It has been found that children with narcolepsy, regardless of pharmaceutical treatment or presence of cataplexy, have higher body mass index (BMIs) [8]. There have been suggestions that these findings are related to a combination of the reduction in basal metabolism and physical activity due to sleepiness [3]. 

There is additional evidence that these patients are at increased risk for various eating disorders. For example, Fortuyn et al. found narcoleptic patients to report irresistible and persistent craving for food, specifically binge eating with lack of control and restrictive actions to correct binging [25]. Eating disorders, such as anorexia/bulimia nervosa, are typically driven by a desire for specific body habitus. There has been some suggestion of fear of becoming fat endorsed by some patients, but in general, this is not the underlying motive for such behaviors in patients with narcolepsy [5,25]. These patterns of behavior require further exploration as hypocretin stimulates appetite [35]. Therefore, a deficiency would be expected to result in decreased food seeking behavior and weight loss. However, fragmented sleep can modify leptin and ghrelin secretion, thus supporting the increased appetite and weight gain, besides the low basal metabolic rate [36].

## 6. Schizophrenia

Schizophrenia and narcolepsy have significant overlap in symptoms including hallucinations, sleep fragmentation, and psychosis (Table 2). In general, hallucinations present in narcolepsy are visual, whereas in schizophrenia they are more so auditory [12]. However, it is not uncommon for hallucinations in narcolepsy to be complex multi-sensory phenomena, which can lead to confusion. Comorbid schizophrenia and narcolepsy has been reported, but is thought to be rare [4] (Table 3). 

## 7. Pathophysiology Overlap

There are only about 70,000 hypocretin cells (HRT-1 and HRT-2), which are concentrated in the lateral hypothalamus. The understood role of hypocretin at this time is in relation to arousal and reward circuitry. Although small in number, the axons of these cells project widely throughout the cortex in varying densities. Hypocretin and dopamine have significant overlap, particularly in the basal forebrain, thalamic paraventricular nucleus, and prefrontal cortex [39]. There are similar overlapping circuits for hypocretin and other monoamines, such as serotonin and norepinephrine. Hypocretin has been shown to have direct excitatory effects on serotonergic neurons, especially in the dorsal raphe nucleus [40]. Similarly, there is a direct excitatory effect on the noradrenergic system, with HRT-1 having five times the excitatory effect of HRT-2 [41]. The understanding of the relationship between HRT and various neurotransmitters is rapidly evolving. This intimate interconnectivity leads to the speculation for a shared pathophysiology for narcolepsy and psychiatric illness, but definitive evidence is still lacking. 

## 8. Conclusions

The presence of psychiatric illness in narcolepsy patients is common. The timeline for development of psychiatric symptoms is poorly defined, which may represent contribution of influencing factors such as age of onset, gender, and duration of illness. There is suggestion that the behavioral phenotype of narcolepsy encompasses various traits of psychiatric disease [28]. Alternative considerations include a secondary development of psychiatric illness, such as depression and anxiety, due to the deleterious effects on reduced quality of life in narcolepsy versus a shared pathophysiology for both narcolepsy and psychiatric disease. 

Narcolepsy is associated with an increased risk for poor quality of life which also results in a high socioeconomic burden. Additionally, it has been found to be associated with a 1.5-fold increase in mortality risk compared to those without narcolepsy [34]. It is unclear how the high burden of co-morbid psychiatric disease contributes to this overall. The presence of persistent depressive symptoms has been shown to be an independent risk factor for impaired quality of life [30]. Excessive daytime sleepiness has also been suggested to increase risk for suicidal ideation, which is amplified in the setting of co-morbid depression [42]. 

A large, systematic, US population–based analysis of medical comorbidities associated with narcolepsy confirmed the findings that there is an excessive prevalence for psychiatric illness. These findings were highlighted by significantly higher psychiatric medication use, psychiatry office visits, and mental illness–related service costs [24]. The consideration for a diagnosis of narcolepsy should be considered in atypical and refractory psychiatric illness. It is important to provide a comprehensive psychiatric evaluation in all patients with narcolepsy to improve identification of co-morbid psychiatric illness and provide appropriate treatment. 

The pharmaceutical treatments used in both narcolepsy and psychiatric illness can lend to further difficulty correctly identifying narcolepsy (Figure 2). On the other hand, a paradoxical effect may occur in some cases that may provide guidance for the correct diagnosis. For instance, use of anti-psychotics in schizophrenia can worsen features of narcolepsy and stimulant therapy for narcolepsy may enhance features of psychosis.

## 9. Future Directions

Future studies should focus on identifying the most effective approach to treating patients with narcolepsy and co-morbid psychiatric illness. The high burden of comorbid disease is debilitating and based on current literature is not being adequately treated. Additionally, the cause for increased mortality in patients with narcolepsy remains unclear. Future studies are needed to clarify if this is a result of narcolepsy as an independent risk factor or the cumulative effect of medical and psychiatric co-morbidities present.

Hypocretin neurons have been identified as a part of the central reward circuitry. Therefore, evaluation of the relationship of HRT deficiency with development of psychiatric symptoms may provide further insight to the underlying pathophysiology. In addition, these findings may also identify unique therapeutic strategies for both narcolepsy and mental illness. 

## Figures and Tables

**Table 1:** Comparison of symptoms of narcolepsy and depression [6,12,28].

Narcolepsy	Depression
Severe Excessive Daytime Sleepiness	Fatigue/lack of energy
Sleep Fragmentation	Sleep Initiation/maintenance difficulties +/- psychosis
Hallucinations (Visual/multi-modal)	Psychomotor agitation/retardation
Cataplexy	Reduced Cognition/Poor school performance
Sleep Paralysis	Withdrawn from friends/family
Negative effect on school/work performance	Guilt
Negative effect on socialization	Appetite changes (weight gain/loss)
Weight gain	Suicide

**Table 2:** Comparison of symptoms of narcolepsy and schizophrenia [4,7,37].

Narcolepsy	Schizophrenia
Excessive Daytime Sleepiness	Excessive Daytime Sleepiness/Mania
Sleep Fragmentation	Sleep initiation or maintenance difficulties
Hallucinations (Visual/multi-modal)	Hallucinations (auditory)
Cataplexy	Catatonia
Sleep Paralysis	PLMD/RLS
Nocturnal Movement Disorders (PLMs)	Reduced REM latency/increased REM density
Reduced REM Latency (SOREMPs)	Social Isolation
Negative effect on socialization	Memory loss, slowness in activity, mental confusion
Negative effect on school/work performance	

**Table 3:** Review of psychiatric disorders in narcolepsy.

Reference	Study Details	ADHD	Depression	Anxiety	Eating Disorders	Schizophrenia	Unclassified Mental Illness	Addictive Behavior
Lecendreux, 2015 [9]	Cross Sectional Survey 108 children with NwC/NwoC </= 18 years old 67 Controls	35.3% NwC 19.7% NwoC 4.8% controls	N/A	N/A	N/A	N/A	N/A	N/A
Modestino, 2013 [11]	Retrospective (ADHD symptoms in childhood)161 adults NwC/NwoC 117 controls	37% Nw/woC	10.55% Nw/woC	N/A	N/A	N/A	N/A	N/A
Lee, 2017 [10]	Case Contro l258 Nw/woC 2580 Controls	8.8% Nw/woC 0.9% controls (*baseline)	32.7% Nw/woC 6.3% control	N/A	N/A	N/A	N/A	N/A
Black, 2017 [24]	Retrospective (medical claims data analysis) 9312 Nw/woC 46559 Controls	N/A	37.9% Nw/woC 13.8% Controls (* mood disorders)	25.1% Nw/woC 11.9% Controls	17.3% Nw/woC 8.4% Controls (*obesity)	N/A	62.3% Nw/woC 31.2% Controls	N/A
Forutyn, 2011 [6]	Case Control 60 Nw/woC 120 Controls	N/A	13% Nw/woC 5% Controls	35% Nw/woC 3% Controls	N/A	N/A	N/A	N/A
Canellas, 2014 [4]	Case series 10 Narcolepsy Patients	N/A	N/A	N/A	N/A	100% overlap	N/A	N/A
Dahmen, 2008 [5]	Case Control 116 Nw/woC 80 Controls	N/A	N/A	N/A	13% Nw/woC 18% Controls	N/A	N/A	N/A
Chabas, 2007 [3]	Case Control 13 Nw/woC 9 Controls	N/A	N/A	N/A	*Eat40 Score* Nw/woC 2x higher than Controls *Bulimia* 46% Nw/woC 11% control	N/A	N/A	N/A
Fortuyn, 2008 [25]	Case Control 60 NwC 120 Controls	N/A	N/A	N/A	Eating Disoder NOS 15–25% NwC 0% controls	Eating Disoder NOS 15–25% NwC 0% controls	N/A	N/A	N/A
Barateau, 2016 [38]	Case Control 710 Controls 243 NwC 116 NwoC 91 IH	N/A	N/A	N/A	N/A	N/A	N/A	*Alcohol* 7.5% NwC 15.2% controls NSD in IH, control, and NwoC *Tobacco* 37.2% NwC 21.7% controls Illicit Drugs NSD in groups

NwC: narcolepsy with cataplexy, NwoC: narcolepsy without cataplexy, Nw/woC: narcolepsy with or without cataplexy, IH: Idiopathic Hypersomnia, NSD no significant differences. N/A: Not applicable.

## References

[1] Thorpy M., Morse A.M. (2017). Reducing the clinical and socioeconomic burden of narcolepsy by earlier diagnosis and effective treatment. Sleep Med. Clin..

[2] Lishman W. (1998). The psychologIcal consequences of cerebral disorder. Organic Psychiatry.

[3] Chabas D., Foulon C., Gonzalez J., Nasr M., Lyon-Caen O., Willer J.-C., Derene J.-P., Arnulf I. (2007). Eating disorder and metabolism in narcoleptic patients. Sleep.

[4] Canellas F., Lin L., Julià M.R., Clemente A., Vives-Bauza C., Ollilla H.M., Chul Hong S., Arboleya S.M., Einen M.A., Faraco J. (2014). Dual cases of type 1 narcolepsy with schizophrenia and other psychotic disorders. J. Clin. Sleep. Med..

[5] Dahmen N., Becht J., Engel A., Thommes M., Tonn P. (2008). Prevalence of eating disorders and eating attacks in narcolepsy. Neuropsychiatr. Dis. Treat..

[6] Fortuyn H.A.D., Lappenschaar G., Furer J.W., Hodiamont P.P., Rijnders C.A., Renier W.O., Buitelaar J.K., Overeem S. (2010). Anxiety and mood disorders in narcolepsy. Gen. Hosp. Psychiatr..

[7] Fortuyn H.A.D., Lappenschaar G., Nienhuis F.J., Furer J.W., Hodiamont P.P., Rijnders C.A., Lammers G.J., Renier W.O., Buitelaar J.K., Overeem S. (2009). Psychotic symptoms in narcolepsy: Phenomenology and a comparison with schizophrenia. Gen. Hosp. Psychiatr..

[8] Kotagal S., Krahn L.E., Slocumb N. (2004). A putative link between childhood narcolepsy and obesity. Sleep Med..

[9] Lecendreux M., Lavault S., Lopez R., Inocente C.O., Konofal E., Cortese S., Franco P., Arnulf P., Dauvilliers Y. (2015). Attention-deficit/hyperactivity disorder (ADHD) symptoms in pediatric narcolepsy: A cross-sectional study. Sleep.

[10] Lee M.J., Lee S.Y., Yuan S.S., Yang C.-J., Yang K.-C., Lee T.-L., Sun C.-C., Shyu Y.-C., Wang L.-J. (2017). Comorbidity of narcolepsy and depressive disorders: A nationwide population-based study in Taiwan. Sleep Med..

[11] Modestino E.J., Winchester J. (2013). A retrospective survey of childhood ADHD symptomatology among adult narcoleptics. J. Atten. Disord..

[12] Vourdas A., Shneerson J., Gregory C., Smith I.E., King M.A., Morrish E., McKenna P.J. (2002). Narcolepsy and psychopathology: Is there an association?. Sleep Med..

[13] Ghanizadeh A. (2013). Agreement between diagnostic and statistical manual of mental disorders, and the proposed DSM-V attention deficit hyperactivity disorder diagnostic criteria: An exploratory study. Compr. Psychiat..

[14] Weinberg W.A., Brumback R.A. (1990). Primary disorder of vigilance: A novel explanation of inattentiveness, daydreaming, boredom, restlessness, and sleepiness. J. Pediatr..

[15] Sultan S., Bertrim S., Kimoff R., Baltzan M. (1998). Syndrome Z: A description of a possible narcolepsy spectrum disorder. Sleep.

[16] Beebe D.W. (2006). Neurobehavioral morbidity associated with disordered breathing during sleep in children: A comprehensive review. Sleep.

[17] Gruber R. (2009). Sleep characteristics of children and adolescents with attention deficit-hyperactivity disorder. Child Adolesc. Psychiatr. Clin..

[18] Hvolby A. (2015). Associations of sleep disturbance with ADHD: Implications for treatment. ADHD Atten. Deficit Hyperact. Disord..

[19] Maski K., Steinhart E., Williams D., Scammell T., Flygare J., McCleary K., Gow M. (2017). Listening to the patient voice in narcolepsy: Diagnostic delay, disease burden, and treatment efficacy. J. Clin. Sleep Med..

[20] Craig S.G., Weiss M.D., Hudec K.L., Gibbins C. (2017). The functional impact of sleep disorders in children with ADHD. J. Atten. Disord..

[21] Alberto K., García-García F. (2013). Current and emerging options for the drug treatment of narcolepsy. Drugs.

[22] Cortese S., Holtmann M., Banaschewski T., Buitelaar J., Coghill D., Danckaerts M., Dittman R.W., Graham J., Taylor E., Sergeant J. (2013). Practitioner review: Current best practice in the management of adverse events during treatment with ADHD medications in children and adolescents. J. Child Psychol. Psychiatr..

[23] Hysing M., Sørensen L., Plessen K., Adolfsdottir S., Lundervold A. (2014). Review: Recommendations for the assessment and management of sleep disorders in ADHD. Evid. Based Ment. Health.

[24] Black J., Reaven N., Funk S., McGaughey K., Ohayon M.M., Guilleminault C., Ruoff C. (2017). Medical comorbidity in narcolepsy: Findings from the burden of narcolepsy disease (BOND) study. Sleep Med..

[25] Fortuyn H.A.D., Swinkels S., Buitelaar J., Renier W.O., Furer J.W., Rijnders C.A., Hodiamont P.P., Overeem S. (2008). High prevalence of eating disorders in narcolepsy with cataplexy: A case-control study. Sleep.

[26] Daniels E., King M.A., Smith I.E., Shneerson J.M. (2001). Health-related quality of life in narcolepsy. J. Sleep Res..

[27] Dauvilliers Y., Paquereau J., Bastuji H., Drouot X., Weil J.S., Viot-Blanc V. (2009). Psychological health in central hypersomnias: The french harmony study. J. Neurol. Neurosurg. Psychiatr..

[28] Fortuyn H.A.D., Mulders P., Renier W., Buitelaar J., Overeem S. (2011). Narcolepsy and psychiatry: An evolving association of increasing interest. Sleep Med..

[29] Zamarian L., Högl B., Delazer M., Hingerl K., Gabelia D., Mitterling T., Brandauer E., Frauscher B. (2015). Subjective deficits of attention, cognition and depression in patients with narcolepsy. Sleep Med..

[30] Vignatelli L., Plazzi G., Peschechera F., Delaj L., D’Alessandro R. (2011). A 5-year prospective cohort study on health-related quality of life in patients with narcolepsy. Sleep Med..

[31] Schwartz S., Ponz A., Poryazova R., Werth E., Boesiger P., Khatami R., Bassetti C.L. (2007). Abnormal activity in hypothalamus and amygdala during humour processing in human narcolepsy with cataplexy. Brain.

[32] Brundin L., Björkqvist M., Petersén Å., Träskman-Bendz L. (2007). Reduced orexin levels in the cerebrospinal fluid of suicidal patients with major depressive disorder. Eur. Neuropsychopharmacol..

[33] Schmidt F.M., Arendt E., Steinmetzer A., Bruegel M., Kratzsch J., Strauss M., Baum P., Hegerl U., Schönknecht P. (2011). CSF-hypocretin-1 levels in patients with major depressive disorder compared to healthy controls. Psychiatr. Res..

[34] Ohayon M.M., Black J., Lai C., Eller M., Guinta D., Bhattacharyya A. (2014). Increased mortality in narcolepsy. Sleep.

[35] Baumann C.R., Bassetti C.L. (2005). Hypocretins (orexins): Clinical impact of the discovery of a neurotransmitter. Sleep Med. Rev..

[36] Garcia-Garcia F., Juárez-Aguilar E., Santiago-García J., Cardinali D.P. (2014). Ghrelin and its interactions with growth hormone, leptin and orexins: Implications for the sleep–wake cycle and metabolism. Sleep Med. Rev..

[37] Taylor S.F., Tandon R., Shipley J.E., Eiser A.S., Goodson J. (1991). Sleep onset REM periods in schizophrenic patients. Biol. Psychiatr..

[38] Barateau L., Jaussent I., Lopez R., Boutrel B., Leu-Semenescu S., Arnulf I., Dauvilliers Y. (2016). Smoking, alcohol, drug use, abuse and dependence in narcolepsy and idiopathic hypersomnia: A case-control study. Sleep..

[39] Deutch A.Y., Bubser M. (2007). The orexins/hypocretins and schizophrenia. Schizophr. Bull..

[40] Liu R.J., van den Pol A.N., Aghajanian G.K. (2002). Hypocretins (orexins) regulate serotonin neurons in the dorsal raphe nucleus by excitatory direct and inhibitory indirect actions. J. Neurosci..

[41] Mieda M., Tsujino N., Sakurai T. (2013). Differential roles of orexin receptors in the regulation of sleep/wakefulness. Front. Endocrinol..

[42] Chellappa S.L., Araújo J.F. (2006). Excessive daytime sleepiness in patients with depressive disorder. Rev. Bras. Psiquiatr..

